# Novel Detection Method for Circulating EGFR Tumor DNA Using Gravitationally Condensed Gold Nanoparticles and Catalytic Walker DNA

**DOI:** 10.3390/ma15093301

**Published:** 2022-05-05

**Authors:** Juneseok You, Chanho Park, Kuewhan Jang, Jinsung Park, Sungsoo Na

**Affiliations:** 1 Department of Mechanical Engineering, Korea University, Seoul 02841, Korea; protrossko1@korea.ac.kr; 2 Division of Foundry, Samsung Electronics, Hwaseong-si 18448, Korea; chanhoo91@korea.ac.kr; 3 School of Mechanical Engineering, Hoseo University, Asan 31499, Korea; kwjang@hoseo.edu; 4 Department of Biomechatronics Engineering, Sungkyunkwan University (SKKU), 2066 Seobu-ro, Suwon 16419, Korea

**Keywords:** circulating tumor DNA, walker DNA, gold nanoparticle, UV-vis absorbance

## Abstract

The detection of circulating tumor DNA is a major challenge in liquid biopsies for cancer. Conventionally, quantitative polymerase chain reactions or next-generation sequencing are used to detect circulating tumor DNA; however, these techniques require significant expertise, and are expensive. Owing to the increasing demand for a simple diagnostic method and constant monitoring of cancer, a cost-effective detection technique that can be conducted by non-experts is required. The aim of this study was to detect the circulating tumor DNA containing the epidermal growth factor receptor (EGFR) exon 19 deletion, which frequently occurs in lung cancer. By applying walker DNA to a catalytic hairpin assembly and using the differential dispersibility of gold nanoparticles, we detected EGFR exon 19 deletion mutant #2 DNA associated with lung cancer. Our sensing platform exhibited a limit of detection of 38.5 aM and a selectivity of 0.1% for EGFR exon 19 wild-type DNA. Moreover, we tested and compared EGFR exon 19 deletion mutants #1 and #3 to evaluate the effect of base pair mismatches on the performance of the said technique.

## 1. Introduction

Liquid biopsy is a next-generation cancer diagnostic technique [1]. Previously, tissue biopsies were conducted to detect cancer [2] and were followed by invasive surgical procedures [3]. Epidermal tissue could be extracted using a needle; however, deep-tissue extraction requires abdominal surgical procedures [4]. This made the process expensive and time-consuming. However, with liquid biopsies, any bodily fluid from the patient, including blood, urine, cerebrospinal fluid, and saliva, can be used to diagnose cancer. Therefore, liquid biopsy is noninvasive as well as cost and time effective [5], making it a useful technique for the early detection of cancer and monitoring cancer transfer [6].

Cell-free DNA (cfDNA) was discovered by Mandel and Metais in 1948 [7]. cfDNA is present in the blood of patients with cancer, and healthy individuals [8]. Although a high concentration of cfDNA can be a sign of cancer, cfDNA alone does not provide conclusive evidence. However, circulating tumor DNA (ctDNA) is conclusive evidence of cancer metastasis [9]. ctDNA is a mutated form of the wild-type cfDNA. Nucleic acid mutations can occur in various ways, such as deletion, exchange, and addition [10,11]. In this study, the epidermal growth factor receptor (EGFR) exon number 19 was selected as the target for cancer detection.

The EGFR ctDNA was mutated by the deletion of 15 nucleic acids at exon 19 [12]. This mutated ctDNA, which is strongly associated with cancer metastasis, was discovered in a patient with lung cancer [13]. The EGFR mutation manipulates the cell growth factor reporter to signal constant cell growth, thereby causing the normal cell to become cancerous [14]. Although the EGFR ctDNA and normal cfDNA are distinguishable via DNA hybridization, the detection of an extremely low level of ctDNA requires an ultrasensitive and highly selective technique [15]. On average, the concentration of cfDNA in blood is 50 fM, which is <1% compared with that of normal cfDNA [16,17]. The target DNA sequence was selected as following deletion types from wild type DNA. The detailed sequence selection is described in Supplementary (Figure S1) [18] Literature studies of EGFR detection was shown in Table S1 [19,20,21,22,23,24,25,26,27].

Catalytic DNA hybridization is a useful tool to detect a low level of target DNA. Commonly used DNA hybridization methods are nucleic acid sequence-based amplification (NASBA) [28,29], rolling circle amplification (RCA) [30,31,32,33,34], strand displacement amplification (SDA) [35,36,37], catalytic hairpin assembly (CHA) [38,39,40], and loop-mediated isothermal amplification (LAMP) [41,42,43,44]. Additionally, colorimetry is a useful method to detect phenomena such as G-quadruplex formation [45,46], Au nanoparticle aggregation [47,48], and chemical color changes [49,50,51,52]. In this study, a combination of catalytic walker DNA and condensed gold nanoparticles (AuNPs) was used to detect EGFR ctDNA. Catalytic walker DNA has several advantages [53]: First, via strand displacement, the catalytic DNA reaction occurs continuously. The walker DNA has two identical functional nucleic acid sites. While one side of the walker DNA is undergoing a reaction, the other side moves to the next DNA. These two reactions are cyclically repeated. Second, to enable walking to the neighboring DNA, this reaction is conducted on a DNA-immobilized surface. Lastly, once the reaction begins, it is unstoppable until it covers the entire surface of the AuNP.

**Figure 1:**
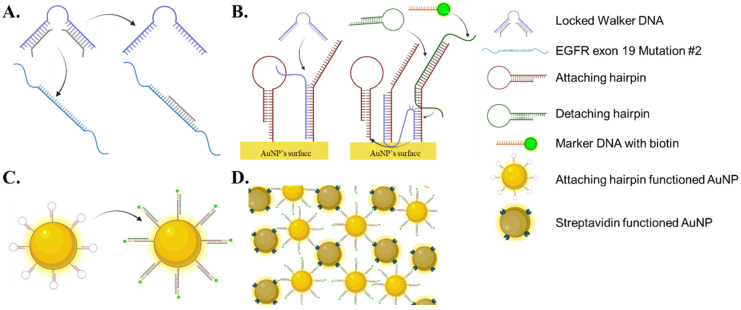
Scheme of working of walker DNA and coupling with the Au NP. (**A**) Locker DNA is bound to walker DNA, until the target DNA (EGFR exon 19 mutant #2) frees the walker DNA by binding to the locker DNA. (**B**) The activated walker DNA opens the attaching hairpin on the AuNP through strand displacement. Following this, the detaching hairpin and marker DNA bind to the attaching hairpin. (**C**) Consequently, biotin molecules are immobilized on the surface of the AuNP. The walker DNA chain reaction continues to the neighboring AuNPs. (**D**) The biotin-immobilized AuNPs bind to the streptavidin AuNPs to produce aggregates.

The detailed procedure is illustrated in Figure 1. Before detecting ctDNA, the attaching hairpin is immobilized on the AuNP. The walker DNA is bound to two locker DNAs. The detaching hairpin, locked walker DNA, and the attaching hairpin functionalized AuNP do not bind to each other. After the addition of the target DNA, the solution is centrifuged to condense the AuNPs. The target DNA takes the locker DNA from the walker DNA. The locker-free walker DNA then opens the attaching hairpin on the AuNP. Subsequently, the detaching hairpin detaches one side of the walker DNA, and the detached site binds to and opens the neighboring attaching hairpin. These steps are cyclically repeated, and the walker DNA travels all over the attaching hairpins on the AuNPs. 

In this study, we utilized walker DNA and hairpin DNA-functionalized AuNPs to detect the EGFR ctDNA using the naked eye and ultraviolet and visible ray (UV-vis) absorbance. Our method achieved a limit of detection (LOD) of 38.5 aM and a selectivity of 0.1% with other types of mutants and normal cfDNA. 

## 2. Materials and Method

### 2.1. Materials

The following materials were purchased from Sigma-Aldrich (St. Louis, MO, USA): sodium chloride (NaCl), tris (2-carboxyethyl) phosphine (TCEP), Tris-ethylene diamine tetra acetic acid (EDTA) buffer solution, ultrapure 10× TBE buffer, ultrapure agarose gel powder, streptavidin, and citrate AuNPs with a diameter of 20 nm. DNAs were purchased from Integrated DNA Technology (Coralville, IA, USA), and all DNAs were purified via HPLC. We used a multi-plate reader (Spectra MAX I 3×, Molecular Devices Corp., Sunnyvale, CA, USA) for measuring UV-vis absorbance of the AuNP solutions. We used a 96-well plate coaster for measuring UV-vis spectra (Corning, NY, USA). 

### 2.2. Preparation of Hairpin DNA and Other DNAs

Attaching hairpin, detaching hairpin, and marker DNAs were designed to have a hairpin structure. The DNA sequences are presented in Table 1. The DNAs were resuspended in a Tris-EDTA solution and 200 mM sodium chloride and heated at 95 °C for 5 min; the DNA solutions were then annealed at room temperature (~25 °C). Locker DNA, walker DNA, and target DNAs were designed to be single-stranded DNA. These DNAs were resuspended in Tris-EDTA, and the locker DNA was conjugated with walker DNA to deactivate walker DNA. 

### 2.3. Fabrication of Attaching Hairpin DNA-Functionalized Gold Nanoparticles and Streptavidin Immobilization

To immobilize the attaching hairpin DNA on an AuNP, a gold-thiol bond was used. The citrate coating on the AuNPs was replaced with thiol DNA by stirring with a magnetic bar for 16 h. The gold solution was centrifuged at 16,000× *g* for 10 min. Except for the sediment of the AuNPs, the transparent solution was replaced with a Tris-EDTA buffer solution. To confirm the stability of the DNA immobilized AuNPs, 800 mM of NaCl was added, and observed change the color of solution. Additional stability and feasibility test results are shown in Supplementary Figure S2.

The streptavidin solution was prepared by mixing with the AuNP solution at a ratio of 1:5. The solution was stirred with a magnetic bar for 16 h at 10 °C. The solution was centrifuged at 16,000× *g* for 10 min; the free streptavidin was removed, and a Tris-EDTA buffer solution was added. 

### 2.4. Preparation of the Control Solution and the Detection Assay

The assay solution consisted of an attaching hairpin-immobilized AuNP solution (60 μL), a detaching hairpin solution (12 μL), a marker DNA solution (3 μL), and a deactivated walker DNA (walker DNA + locker DNA) solution (6 μL), which had two locker DNAs on both sides. The total sodium concentration was fixed at 800 mM. Various assay solutions were prepared with different concentrations of deactivated walker DNA to estimate the LOD and evaluate the sensitivity. For the detection group, target DNA (EGFR exon 19 deletion mutant) was added to the assay solution at various concentrations. For control group, target DNA was not added.

The target DNA was detected by adding it to the prepared assay solution. After incubating the target DNA with the assay solution for 20 min, the attaching hairpin-immobilized AuNP solution (60 μL) was added and centrifuged at 6000× *g* for 10 min. The solution was maintained at 37 °C for 3 h to allow activated walker DNA reaction. Afterwards, the streptavidin-immobilized AuNP solution was added, and the solution was centrifuged at 2000× *g* for 5 min. After 6 h, 100 μL of the supernatant was extracted and its UV-vis spectrum was measured.

### 2.5. Design of the Detection Experiment

The UV-visible spectra of the samples were measured from 400 to 700 nm in a 96-well plate (Figures S3 and S4). Based on the concentration of template, we prepared corresponding control solutions and detection solutions at various concentrations. We compared the UV-vis absorbance spectra of assay solution, which did not contain the target DNA, and the detection solution, which contained it. We analyzed the area of the UV absorbance spectrum of each sample using the Peakfit software. From the area value, we calculated the detection signal from Equation (1). All the area parameters were obtained from the Peakfit software [54,55]. The area value was calculated by Gaussian curve fit and area integration.

(1) [{(C.S.)−(D.S.)}×100]⁄(C.S.) (%) 

where C.S. and D.S. represent the UV-vis absorbance intensity of the control solution and detection solution, respectively.

### 2.6. Gel Electrophoresis Proof

The electrophoresis of the walker DNA was performed using 2% agarose gels in 1× TBE buffer. Each DNA solution was dissolved in Tris-EDTA buffer, and the final concentration of the sodium ion was set at 200 mM. One DNA solution was added to each lane, and ladder DNA (50 bp) was added to the far-left lane as a reference. In each sample, the total concentration of DNA was 10 μM, and the volume of the solution was 10 μL. The fluorescent detection solution and DNA solution were mixed at a ratio of 1:3. The voltage was set to 100 mV, and running time was 20 min. After separation, the gel was photographed by a CCD camera (Samsung Electronics Co., Ltd., Suwon, Korea) in a UV-vis light box.

## 3. Results and Discussion

### 3.1. Detection of EGFR Mutant DNA

To quickly detect the target DNA, the solution was centrifuged at 2000× *g* for 5 min. In case of a positive reaction, the AuNPs were coupled, and therefore, settled at the bottom. In case of a negative reaction, the AuNPs were not coupled, and therefore, remained suspended in the solution. This difference in dispersibility, was reflected in a difference in UV absorbance at 520 nm. The control solution had a high absorbance, while the positive solution had a low absorbance. The aggregated nanoparticles were precipitated; however, the color of the solution remained red since the distance between the AuNPs was sufficiently large. The distance between the AuNPs was 70 base pairs of DNA [19,20]. Therefore, we compared the upper part of the solution after extraction and measured the absorbance spectrum instead of the peak shift [55]. The area of the peak at 524 nm indicated the purity of single AuNPs and the existence of a number of AuNPs. A sharp and large area was indicative of a single AuNP spectrum. The presence of the EGFR ctDNA was determined based on the difference in the UV spectrum area between the control solution and positive solution.

### 3.2. Mechanism of the Walker DNA

**Figure 2:**
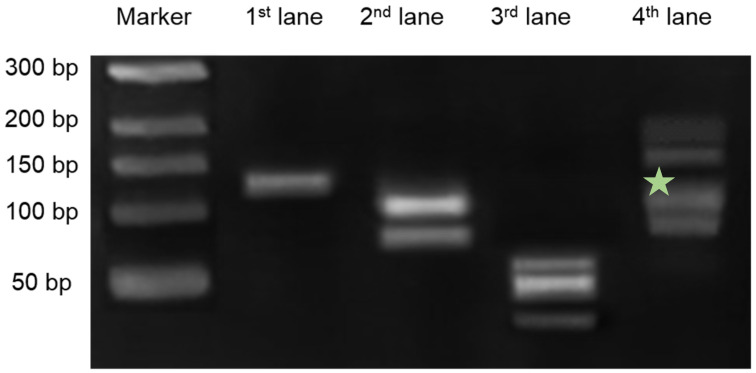
Results of gel electrophoresis of Walker DNA. Gel electrophoresis of DNA structures. Lane 1. locked Walker DNA; Lane 2. unlocked Walker DNA and target DNA; Lane 3. separated attaching hairpin, detaching hairpin, and marker DNA; Lane 4. target DNA + locker DNA; walker DNA fully assembled DNA and target DNA. The green star denotes the position of Assembled DNA (attaching hairpin + detaching hairpin + marker DNA).

We evaluated the DNA chain reaction through gel electrophoresis. As shown in Figure 2 and Figure S5, we loaded the solution in the order of the steps of the detection process. The locked walker DNA is visible at the top in the 1st lane and the target DNA is visible in the 2nd lane. On detachment of the locker DNA from the walker DNA, a lower intensity band corresponding to the walker DNA appeared on the gel. Locker DNA bound to the target DNA is upper band. The 3rd lane shows the bands corresponding to attaching hairpin, detaching hairpin, and marker DNA from the top. The attaching hairpin, detaching hairpin, and marker DNA were mixed; however, there was no hybridization reaction between the hairpins and the marker DNA. The 4th lane shows a mixture of the unlocked walker DNA (2nd lane) and all the DNA of the 3rd lane. This lane has a considerably higher molecular weight band, which indicates that an assembled DNA structure (green star: attaching hairpin + detaching hairpin + marker DNA) was formed because of the activation of the walker DNA. Above 150 bp, it does not show a single band but two bands. The upper band is the walker DNA binding to the two attaching hairpins. The lower band is the fully assembled DNA of the attaching hairpin, detaching hairpin, and marker DNA, which was the end product.

### 3.3. Redispersion Time of the AuNPs

**Figure 3:**
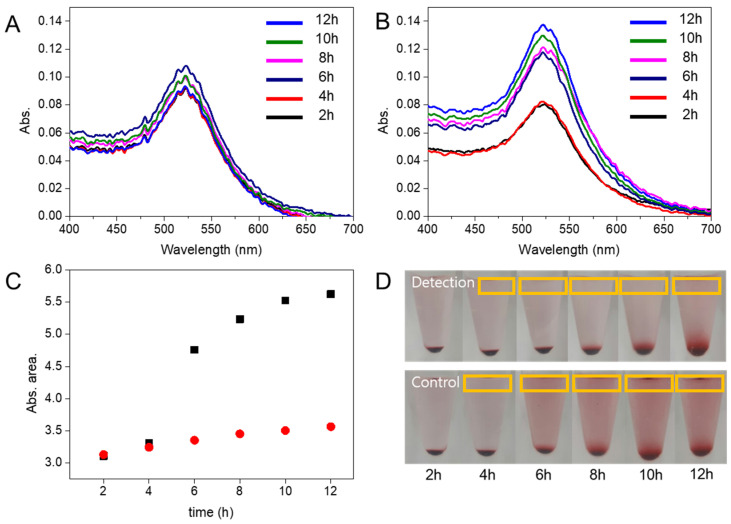
Real-time detection of EGFR exon 19 deletion mutant #2. UV-vis absorbance spectrum (**A**) detection experiment, (**B**) control experiment and relatively. (**C**) area of UV-vis absorbance spectrum (black square: control experiment; red dot: detection experiment), and (**D**) centrifuge cell picture, according to re-dispersion time. The concentration of EGFR mutant DNA was 3.85 × 10^−1^ M.

We measured the UV-vis absorbance to evaluate the redispersion time of the condensed AuNPs. We prepared the same control and detection solutions for each concentration every 2 h and extracted the supernatant solution (Orange box, Figure 3D) based on the time interval. As shown in Figure 3, there was a difference between the spectrum of the control solution and that of the detection solution after 6 h. Figure 3C shows the area of each UV-vis spectrum. The difference in area was evident after 6 h. In the solution, the dispersed AuNPs were free nanoparticles, and the non-dispersed AuNPs were aggregated nanoparticles. Despite the electrostatic repulsive forces of the DNA on the AuNPs, the streptavidin–biotin bonding was strong enough to aggregate the AuNPs.

Since there was no target DNA in the control solution, the AuNPs were dispersed. However, in the detection solution, the target DNA released the walker DNA to produce the DNA complex with biotin at one end, forming streptavidin-biotin-bound AuNP aggregates. Since the target DNA was not present in the control solution, the free marker DNA could bind to the streptavidin AuNPs, thereby resulting in stable streptavidin AuNPs.

### 3.4. Limit of Detection of EGFR Exon 19 Mutant #2

As mentioned above, we tested various concentrations of the target ctDNA, from 3.85 × 10^−1^ nM to 3.85 × 10^−8^ nM. Therefore, we constructed a two-dimensional set for the detection experiment (Figures S3 and S4). The red boxes in Figures S3 and S4 form a diagonal line. It included four samples: the lower control (upper left), lower detection (upper right), higher control (lower left), and higher detection solutions (lower right). Using Equation (1), we determined values of the areas for all the concentrations of the control and detection solutions. The diagonal line represents the dynamic concentration, and shows notable results compared to those obtained in the other experimental sets. Using this platform, any concentration of target DNA can be detected by preparing all the concentrations of the template. We identified the concentration of the target ctDNA by determining the critical dynamic line.

**Figure 4:**
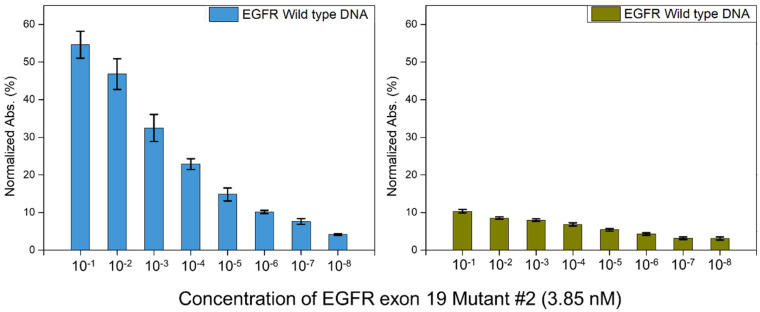
Detection of EGFR exon 19 deletion mutant #2. Detection of EGFR exon 19 wild type DNA (green) and EGFR exon 19 deletion mutant #2 DNA (blue). The exact value of detection of EGFR exon 19 wild type DNA (green) is 10.3% ± 0.4%, 8.5% ± 0.3%, 7.9% ± 0.3%, 6.8% ± 0.4%, 5.4% ± 0.3%, 4.2% ± 0.3%, 3.1% ± 0.3%, and 3.1% ± 0.3%, and the exact value of detection of EGFR exon 19 deletion mutant #2 DNA (blue) is 54.5% ± 3.5%, 46.8% ± 4.0%, 32.4% ± 3.6%, 22.9% ± 1.4%, 14.8% ± 1.7%, 10.1% ± 0.4%, 7.6% ± 0.7%, and 4.1% ± 0.2% in order of the concentrations of template and target DNA. Each concentration has been tested at least three times.

To determine the exact LOD for this platform, we compared the solutions containing mutant DNA with those containing the EGFR wild-type DNA. The blood of a cancer patient contains both mutant ctDNA and wild-type cfDNA. Therefore, the control experiment was set for the detection of wild-type DNA. In Figure 4, the green bars represent the absorbance of wild-type DNA, and the blue bars represent the absorbance of EGFR exon 19 deletion mutant #2 DNA. Each experiment was performed at least three times, over a target DNA concentration range of 3.85 × 10^−1^ nM to 3.85 × 10^−8^ nM. At high concentrations of mutant DNA, a large difference between the wild-type DNA and mutant DNA detection signals was observed. Therefore, following the principle of LOD, we adopted a three-standard deviation (3SD) criterion. The control signal should be the lower template of 3.85 × 10^−8^ nM, which was 3.1% ± 0.3%. The LOD was set to 4.2%. Therefore, using this platform, we could detect 3.85 × 10^−7^ nM (38.5 aM) of the target DNA, which is a remarkably low LOD compared to those of other detection platforms [55,56,57,58,59].

The LOD, as shown in Figure 4, also indicated a higher selectivity for wild-type DNA. For mutant DNA detection, 3.85 × 10^−7^ nM and 3.85 × 10^−6^ nM of the target DNA had selectivity values of 10.1% ± 0.42% and 7.6% ± 0.7 %, respectively, and for wild-type DNA detection, 3.85 × 10^−1^ nM and 3.85 × 10^−3^ nM of the target DNA had selectivity values of 10.3% ± 0.4% and 7.9% ± 0.3%, respectively. The magnitude of the order difference between mutant and wild-type DNA was ×100,000 and ×10,000, respectively, but with similar signals. Therefore, a 1000× concentration of wild-type DNA was distinguishable from that of mutant DNA, which corresponded to a selectivity of 0.1%.

### 3.5. Detection of Other Types of ctDNA

We evaluated the unlocking of the walker DNA with wild-type DNA and EGFR exon 19 mutants #1 and #3. The activation of walker DNA is the first step in the working of this platform. Therefore, the results of the unlocking of walker DNA were compared with the results of detection. In Figure S6, wild-type DNA and mutant #3 DNA exhibited a lower band line. However, mutant #1 and mutant #2 DNA exhibited considerably higher and similar band lines. The mutant #2 DNA and mutant #1 DNA differed by a single base pair in their sequences; however, the wild-type DNA and mutant #3 DNA differed by 15 base pairs and 6 base pairs, respectively. All the mutant DNA had potential binding sites in their sequences.

**Figure 5:**
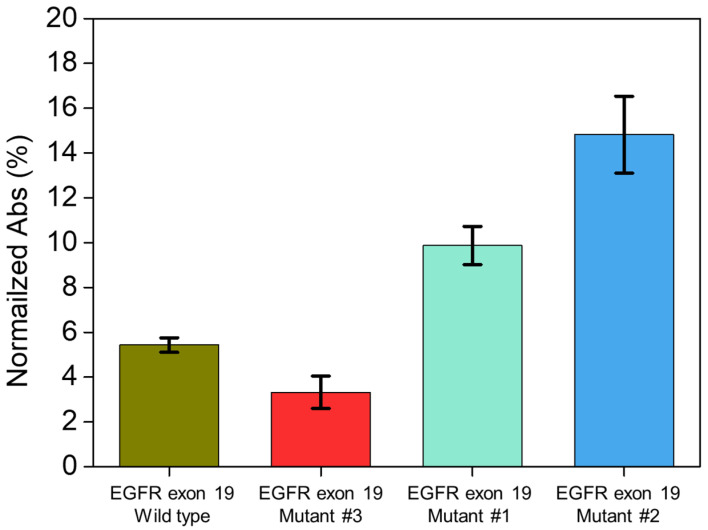
Selectivity experiment of EGFR exon 19 mutants and wild type DNA. The exact value is 5.4% ± 0.3%, 3.3% ± 0.7%, 9.8% ± 0.8%, and 14.8% ± 1.7% for wild-type, mutant #3, mutant #1 and mutant #2, respectively. The concentration of the DNA was 3.85 × 10^−5^ nM. Each bar data has been tested at least three times.

The detection experiment was conducted on the wild-type, mutant #3, mutant #1, and mutant #2 DNA, as shown in Figure 5. The target DNA concentration was fixed at 3.85 × 10^−5^ nM, and the measured values were 5.4% ± 0.3% for the wild-type, 3.3% ± 0.7% for mutant #3, 9.8% ± 0.8% for mutant #1, and 14.8% ± 1.7% for mutant #2. As mutant #1 DNA had a single base pair mismatch with the templates, it exhibited a high intensity signal similar to that of mutant #2 DNA. Conversely, the wild-type and mutant #3 DNA exhibited low intensity signals.

## 4. Conclusions

The detection of EGFR ctDNA in the blood is crucial for the early detection of cancer. In this study, we used catalytic walker DNA and streptavidin–biotin coupled AuNPs for detecting EGFR ctDNA. We detected ctDNA through walker DNA-based gravitational condensation and dispersion of AuNPs. Through this process, this sensing platform achieved a LOD of 38.5 aM and a selectivity of 0.1% for mutant DNA, compared with those achieved for EGFR wild-type DNA. However, the limitation of this platform is the inefficient detection of a single base pair mismatched DNA sequence, as observed in case of mutant #1. In the future, we will attempt to address this limitation.

## Figures and Tables

**Table 1:** The oligonucleotides used in this work.

Name	Sequences
Attaching hairpin	5′ Thiol- GAT TGT GAG ATG TCT TGA CCA TGT TGA GAC TAT CAA GAC ATC TCC—3′
Detaching hairpin	5′ ACA YCY CCY CYA CAC ATG TCT YGA CGA CGG GA -3′
Locker DNA	5′ TGG CTT TCG GAG ATG TCT TGA TAG CGA CGG GA -3′
Walker DNA	5′ CTA TCA AGA CAT CTC CTC ACA ATC CAT CTG TGG TAT CAT CTA TGT ATT CTA TCA AGA CAT CTC CTC ACA ATC 3′
Marker DNA	5′ biotin TEG- TCC ATC CAT GTT GCA CCA GGT AGA TGT 3′
Mutant #1 (84 mer)	5′ GGA CTC TGG ATC CCA GAA GGT GAG AAA GTT AAA ATT CCC GTC GCT ATC AAA ACA TCT CCG AAA GCC AAC AAG GAA ATC CTC GAT 3′
Mutant #2 (84 mer)	5′ GGA CTC TGG ATC CCA GAA GGT GAG AAA GTT AAA ATT CCC GTC GCT ATC AAG ACA TCT CCG AAA GCC AAC AAG GAA ATC CTC GAT 3′
Mutant #3 (90 mer)	5′ GGA CTC TGG ATC CCA GAA GGT GAG AAA GTT AAA ATT CCC GTC GCT ATC AAG GAA GCA ACA TCT CCG AAA GCC AAC AAG GAA ATC CTC GAT 3′
Wild type (99 mer)	5′ GGA CTC TGG ATC CCA GAA GGT GAG AAA GTT AAA ATT CCC GTC GCT ATC AAG GAA TTA AGA GCA ACA TCT CCG AAA GCC AAC AAG GAA ATC CTC GAT 3′

## Data Availability

Data available on request due to restrictions eg privacy or ethical.

## Supplementary Materials

The following are available online at https://www.mdpi.com/article/10.3390/ma15093301/s1, Figure S1: Schematic image of EGFR exon 19 deletion types with codon name and number, Figure S2: Detailed electrophoresis band line explanation, Figure S3: 800 mM of NaCl precipitation test, Figure S4: 96-well plate picture of detection of EGFR exon 19 mutation #2 DNA, Figure S5: UV absorbance spectrum plot of detection of EGFR exon 19 mutation #2, Figure S6: Agarose gel electrophoresis test of selective activation of walker DNA, Table S1: Literature studies of detection of EGFR DNA.
