# Supplementary material for: Novel Detection Method for Circulating EGFR Tumor DNA Using Gravitationally Condensed Gold Nanoparticles and Catalytic Walker DNA
Source: Materials (Basel). 2022 May 5;15(9):3301. doi: 10.3390/ma15093301 (PMC9101948; doi:10.3390/ma15093301)
Supplement: Supplementary file 1 [file materials-15-03301-s001.zip › materials-1637607-supplementary.pdf]

materials

Supplementary

Novel Detection Method for Circulating EGFR Tumor DNA Using Gravitationally Condensed Gold Nanoparticles and Catalytic Walker DNA

Juneseok You, Chanho Park, Kuewhan Jang, Jinsung Park * and Sungsoo Na *

Mutant DNA/wild type DNA sequence selection

In the National center of biotechnology information (NCBI), we find EGFR exon 19 complete sequences. It is reported that the clinical evolution of a patient's disease concerning EGFR mutational assessment. Pisapia. P et al. found an EGRF mutation exon 19 in a lung cancer patient. A next-generation sequencing (NGS) inspection found 15 mer deletions at exon 19 (E746 to A750). We selected mutants #1 and #2 as E746-A750.

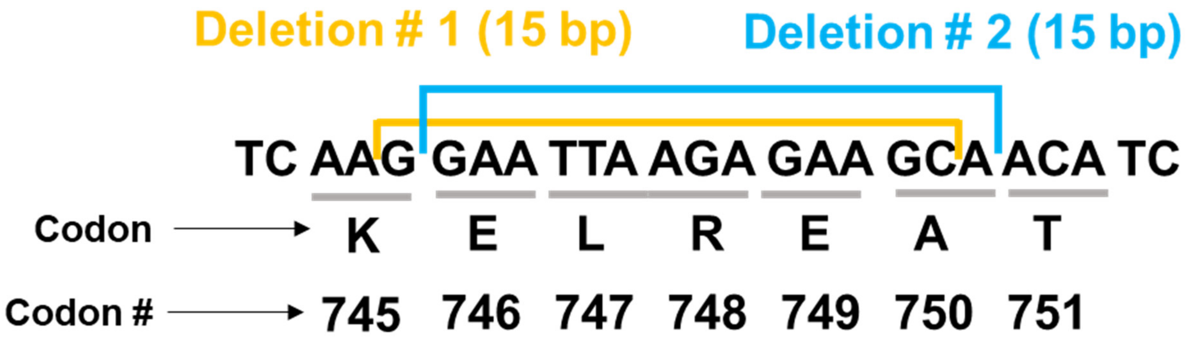

Figure S1. Schematic image of EGFR exon19 deletion types with codon name and number.

The exon 19 deletion E746-A750 occurred, including G and ahead of A (Deletion #2, Cyon). The same deletion is possible as Deletion #1 (orange). The sequence deletion of Deletion #1 and Deletion #2 results in a single sequence variant. Therefore, the deletion E746-A750 includes two types of deletions. According to the reported exon 19 deletions, the significant deletion is mutant #2 (Deletion #2, Cyon). However, mutant #1 (Deletion #1, orange) could exist.

Mutant #3 was selected by having the most similar sequences to the wild-type DNA. In other words, mutant #3 is the shortest deletion. Because of the similarity to wild-type DNA, mutant #3 could be the most challenging variant to be distinguished. That is the reason that we chose mutant #3.

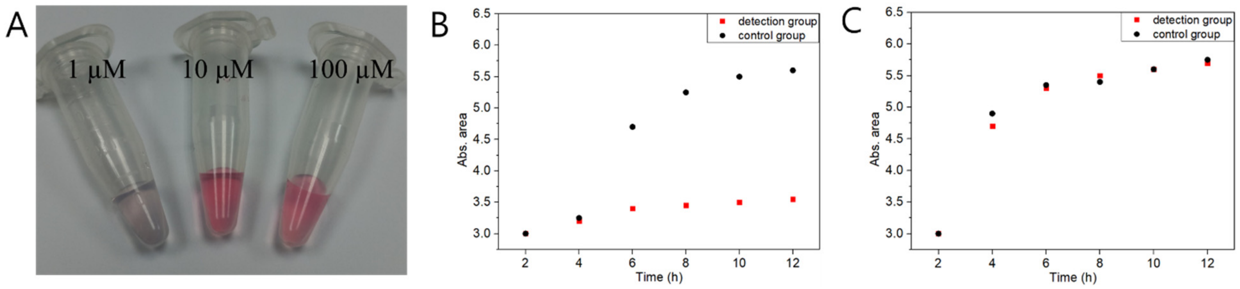

Figure S2. A. 800 mM of NaCl precipitation test. (1 µM, 10 µM, and 100 µM of attaching hairpin DNA immobilized Au nanoparticle). **B and C** represents detection and control experiment using 10 µM and 100 µM of attaching hairpin DNA immobilized Au nanoparticle.

Stability test of AuNPs

One µM of attaching hairpin DNA functioned Au nanoparticle was precipitated in the 800 mM of NaCl, and 10 µM and 100 µM of the sample was well dispersed. Then, we performed the EGFR detection protocol. The target DNA concentration was 3.85 nM. The detection and control group of 100 µM samples was dispersed after 2 hours. Only the 10 µM sample showed the difference.

The total detection time was elongated when DNA was immobilized too much on the Au nanoparticle. Because the catalytic walker DNA reacts, too much DNA attaches at one Au nanoparticle. On the other hand, when DNA was immobilized less on the Au nanoparticle, it caused a lack of stability.

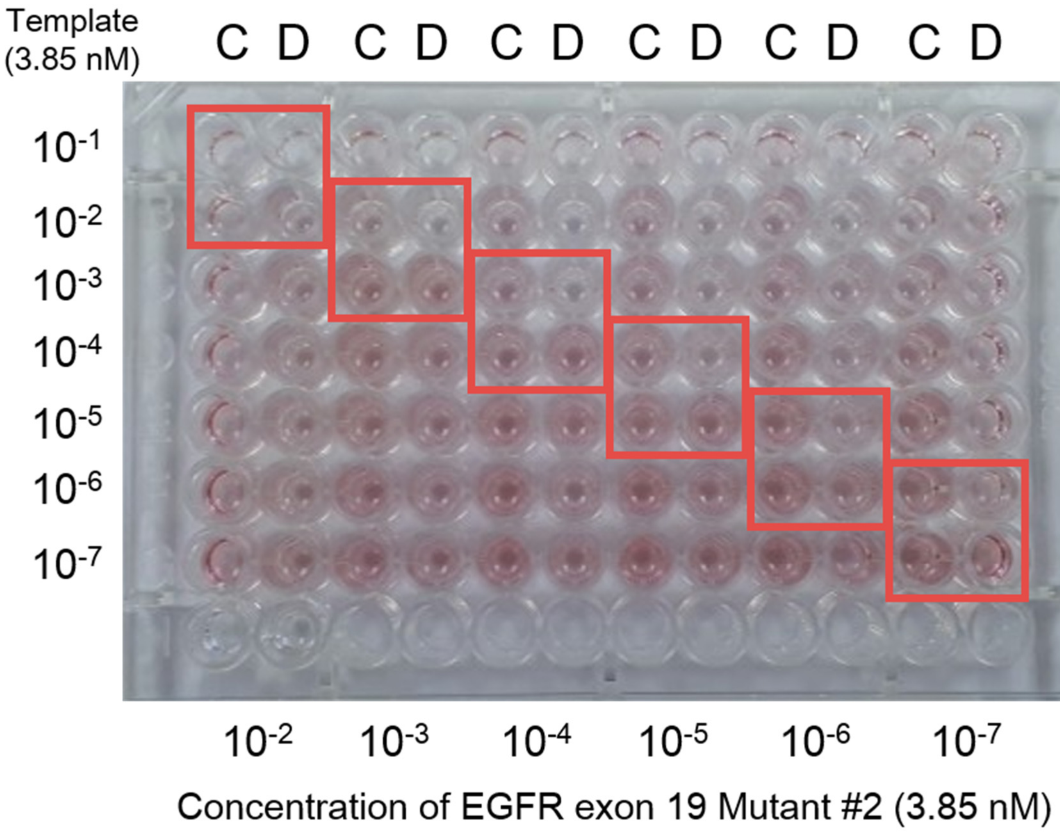

Figure S3. 96-well plate picture of detection of EGFR exon 19 mutation #2 DNA. C and D on the top represents control and detection solution. The red boxes in the plate means diagonal line and detection point.

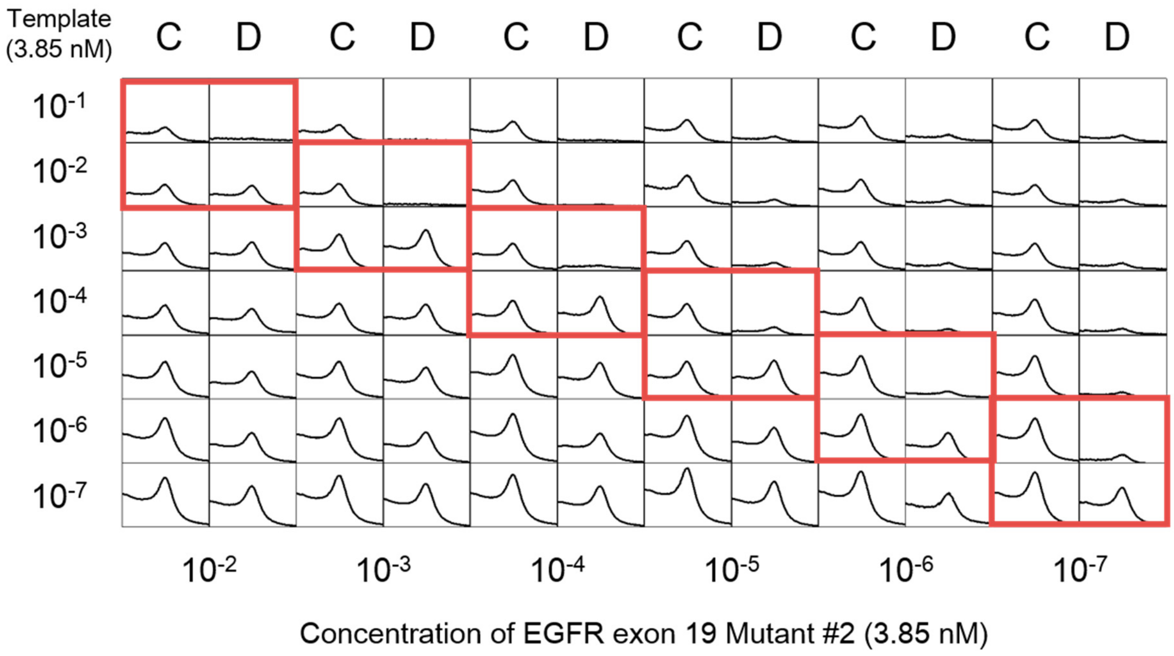

Figure S4. UV absorbance spectrum plot of detection of EGFR exon 19 mutation #2. C and D on the top represents control and detection solution. The red boxes in the plate means diagonal line and detection point

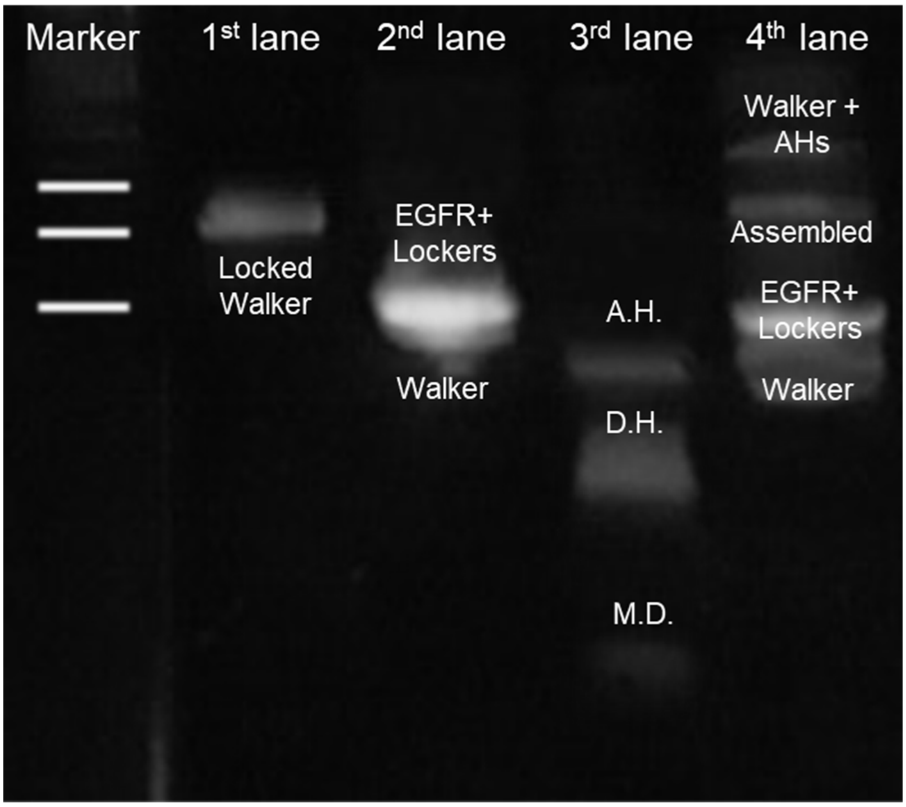

Figure S5. Detailed electrophoresis band line explanation.

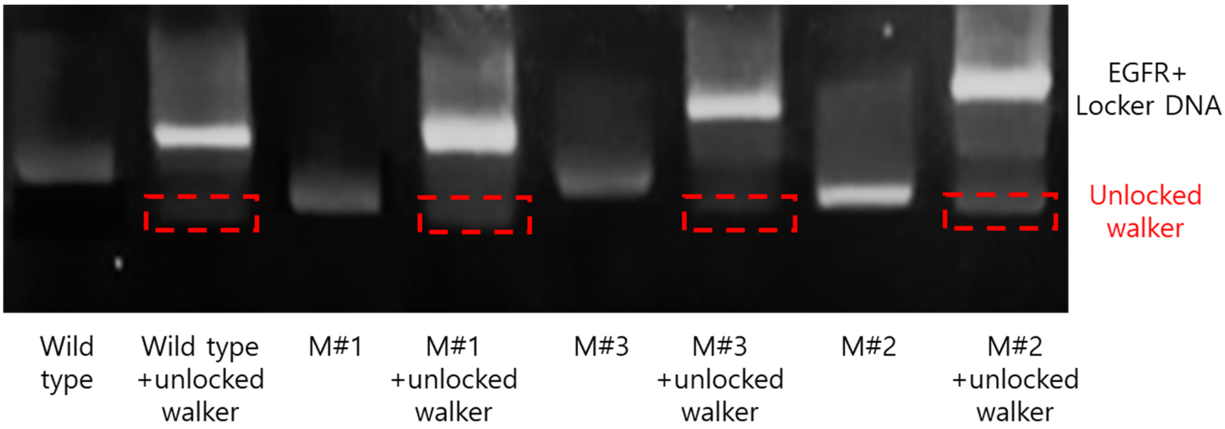

Figure S6. Agarose gel electrophoresis test of selective activation of walker DNA. The concentration of each DNA solution was set at 2μM.

Table S1. Literature studies of detection of EGFR DNA.

	Method	LOD	selectivity
C. Park. et. al (2018)[1]	Catalytic hairpin assembly and gold nanoparticle colorimetry	7.7 fM	1%
C. Park. et. al (2019)[2]	Double amplified colorimetric detection of DNA using gold nanoparticles, enzymes, and a catalytic hairpin	3.1fM	1%
W. et. al (2020)[3]	Tetrahedral DNA nanostructure-decorated electrochemical platform	30 pg	0.1%
H. Yu et. al (2021)[4]	Alleles using the AsCas12a double-stranded DNA trans-cleavage activity	10 aM	0.1%
C Mei et. al (2021)[5]	CRISPR/Cas9 cleavage triggered ESDR (entropy-driven strand displacement reaction) based on a 3D graphene/AuPtPd nanoflower biosensor	0.13pM	~0.1%
X. Mao et. al. (2019)[6]	DNAzyme-functionalized hydrogel	0.32pM	~0.1%
Y. Hu et. al. (2021)[7]	Mn2+-mediated magnetic relaxation switching for direct assay	340 pM	0.1%
O. G. et. al. (2019) [8]	Express and portable label-free DNA detection and recognition with SERS platform based on functional Au grating	10 fM	Not mentioned
X. Yang et. al. (2021)[9]	Electrochemiluminescence resonance energy transfer biosensor	2.3 aM	Not mentioned
**This work**	Catalytic walker DNA and condensed Au nanoparticle redispersion	38.5 aM	0.1%

References

1. Park, C.; Song, Y.; Jang, K.; Choi, C.-H.; Na, S.J.S.; Chemical, A.B. Target switching catalytic hairpin assembly and gold nanoparticle colorimetric for EGFR mutant detection. 2018, 261, 497-504.
2. Park, C.; Park, H.; Lee, H.J.; Lee, H.S.; Park, K.H.; Choi, C.-H.; Na, S.J.M.A. Double amplified colorimetric detection of DNA using gold nanoparticles, enzymes and a catalytic hairpin assembly. 2019, 186, 1-8.
3. Wang, X.; Wu, J.; Mao, W.; He, X.; Ruan, L.; Zhu, J.; Shu, P.; Zhang, Z.; Jiang, B.; Zhang, X.J.A. A tetrahedral DNA nanostructure-decorated electrochemical platform for simple and ultrasensitive EGFR genotyping of plasma ctDNA. 2020, 145, 4671-4679.

4. Lee Yu, H.; Cao, Y.; Lu, X.; Hsing, I.M. Detection of rare variant alleles using the AsCas12a double-stranded DNA trans-cleavage activity. Biosensors and Bioelectronics 2021, 189, 113382, doi:https://doi.org/10.1016/j.bios.2021.113382.
5. Chen, M.; Wu, D.; Tu, S.; Yang, C.; Chen, D.; Xu, Y. CRISPR/Cas9 cleavage triggered ESDR for circulating tumor DNA detection based on a 3D graphene/AuPtPd nanoflower biosensor. Biosensors and Bioelectronics 2021, 173, 112821, doi:https://doi.org/10.1016/j.bios.2020.112821.
6. Mao, X.; Pan, S.; Zhou, D.; He, X.; Zhang, Y. Fabrication of DNAzyme-functionalized hydrogel and its application for visible detection of circulating tumor DNA. Sensors and Actuators B: Chemical 2019, 285, 385-390, doi:https://doi.org/10.1016/j.snb.2019.01.076.
7. Hu, Y.; Guo, X.; Gu, P.; Luo, Q.; Song, Y.; Song, E. Mn2+-mediated magnetic relaxation switching for direct assay of ctDNA in whole blood via exonuclease III assisted amplification. Sensors and Actuators B: Chemical 2021, 330, 129340, doi:https://doi.org/10.1016/j.snb.2020.129340.
8. Guselnikova, O.; Postnikov, P.; Pershina, A.; Svorcik, V.; Lyutakov, O. Express and portable label-free DNA detection and recognition with SERS platform based on functional Au grating. Applied Surface Science 2019, 470, 219-227, doi:https://doi.org/10.1016/j.apsusc.2018.11.092.
9. Yang, X.; Liao, M.; Zhang, H.; Gong, J.; Yang, F.; Xu, M.; Tremblay, P.-L.; Zhang, T. An electrochemiluminescence resonance energy transfer biosensor for the detection of circulating tumor DNA from blood plasma. iScience 2021, 24, 103019, doi:https://doi.org/10.1016/j.isci.2021.103019.
